# An enlarged azygos vein traversing an azygos lobe reveals a connection between the inferior vena cava and azygos vein

**DOI:** 10.1002/rcr2.506

**Published:** 2019-11-27

**Authors:** Hiroyuki Miura, Shinichi Goto, Jun Miura, Keisei Tachibana, Ryota Tanaka, Haruhiko Kondo

**Affiliations:** ^1^ Department of Thoracic Surgery Akiru Municipal Medical Center Tokyo Japan; ^2^ Department of Respirology Akiru Municipal Medical Center Tokyo Japan; ^3^ Department of Surgery Kyorin University School of Medicine Tokyo Japan

**Keywords:** Azygos connection of the inferior vena cava, azygos fissure, azygos lobe, azygos vein, malformation

## Abstract

We report a rare case where the inferior vena cava connected to the azygos vein, a diagnosis triggered by observation of an enlarged azygos vein traversing an azygos fissure. A 16‐year‐old male patient presented with an abnormal shadow on chest X‐ray. Chest computed tomography showed an enlarged azygos vein connecting to the inferior vena cava, with no other connection to the atrium. There were no associated malformations. The patient remains alive and has been asymptomatic for the past two years. If the flow through the connection was to be interrupted during the course of thoracic or abdominal surgery, this would invariably prove to be fatal. In addition, this abnormality prevents direct access to the atrium on attempting interventional radiology via the inferior vena cava, for example, during ablation. When an azygos lobe is identified on a chest X‐ray, a prominent solid structure traversing it may represent an engorged azygos vein with an anomalous course.

## Abstract 2

We report a rare case of the inferior vena cava connecting to the azygos, in a 16‐year‐old male patient who presented with an abnormal shadow on chest X‐ray. The azygos vein was enlarged and ran through an azygos fissure, inviting the diagnosis.

## Graphical abstract

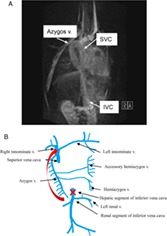

## Introduction

An azygos connection of the inferior vena cava is a rare condition, formed during embryonic development when the renal segment of the inferior vena cava does not connect to the hepatic segment 1. The blood flow of the lower body then drains into the superior vena cava through the right supracardinal vein (the azygos vein). The azygos vein migrates over the apex of the right lung to the mediastinum during the embryonic period. If this migration is delayed, the azygos vein divides the right upper lobe, forming an azygos lobe [2].

We report a rare case of the inferior vena cava connecting to the azygos, in which the azygos vein was enlarged and ran through an azygos fissure, inviting the diagnosis.

## Case Report

**Figure 1:**
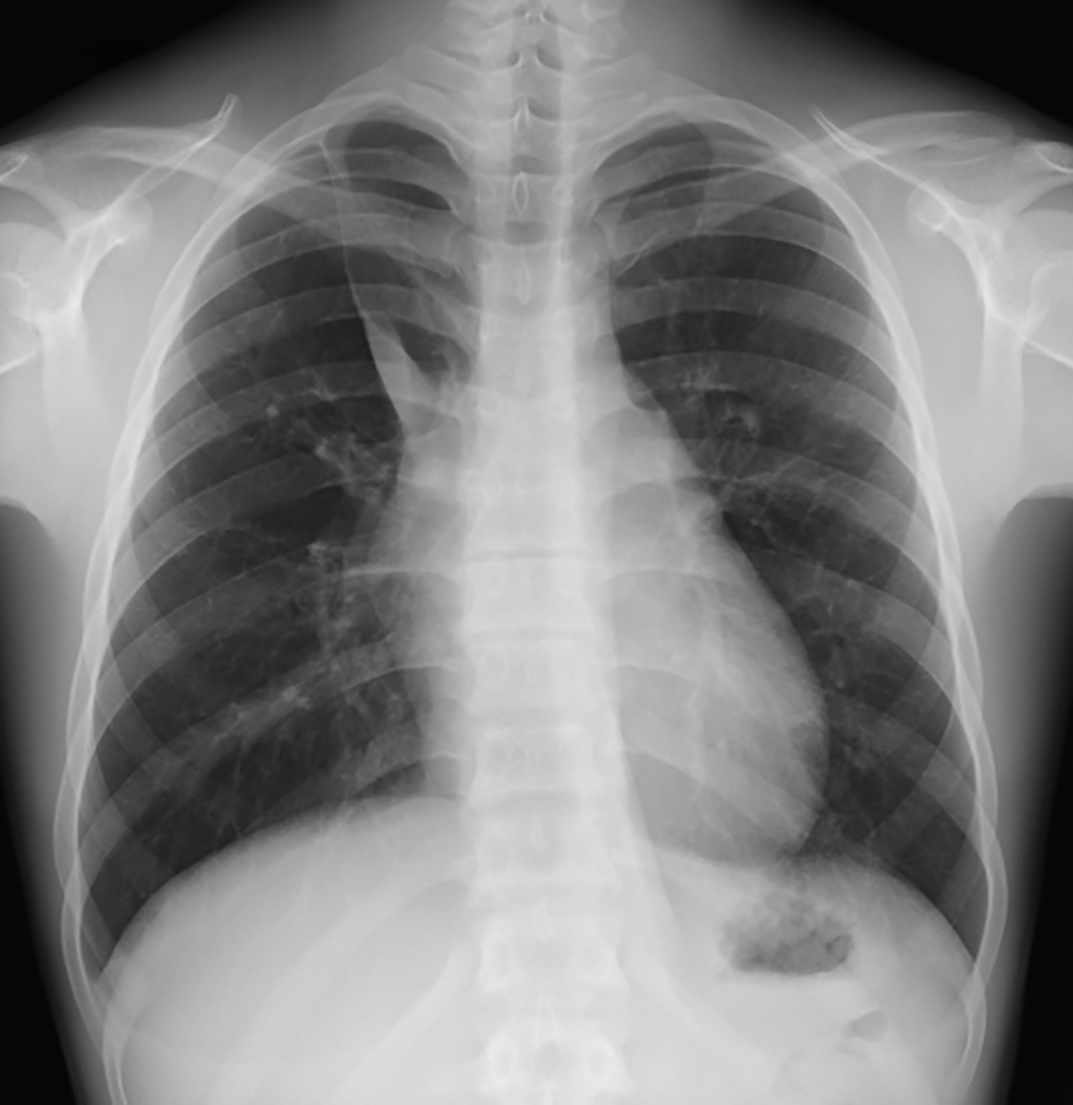
Chest X‐ray shows cardiomegaly and a sickle‐shaped shadow extending towards the mediastinum.

**Figure 2:**
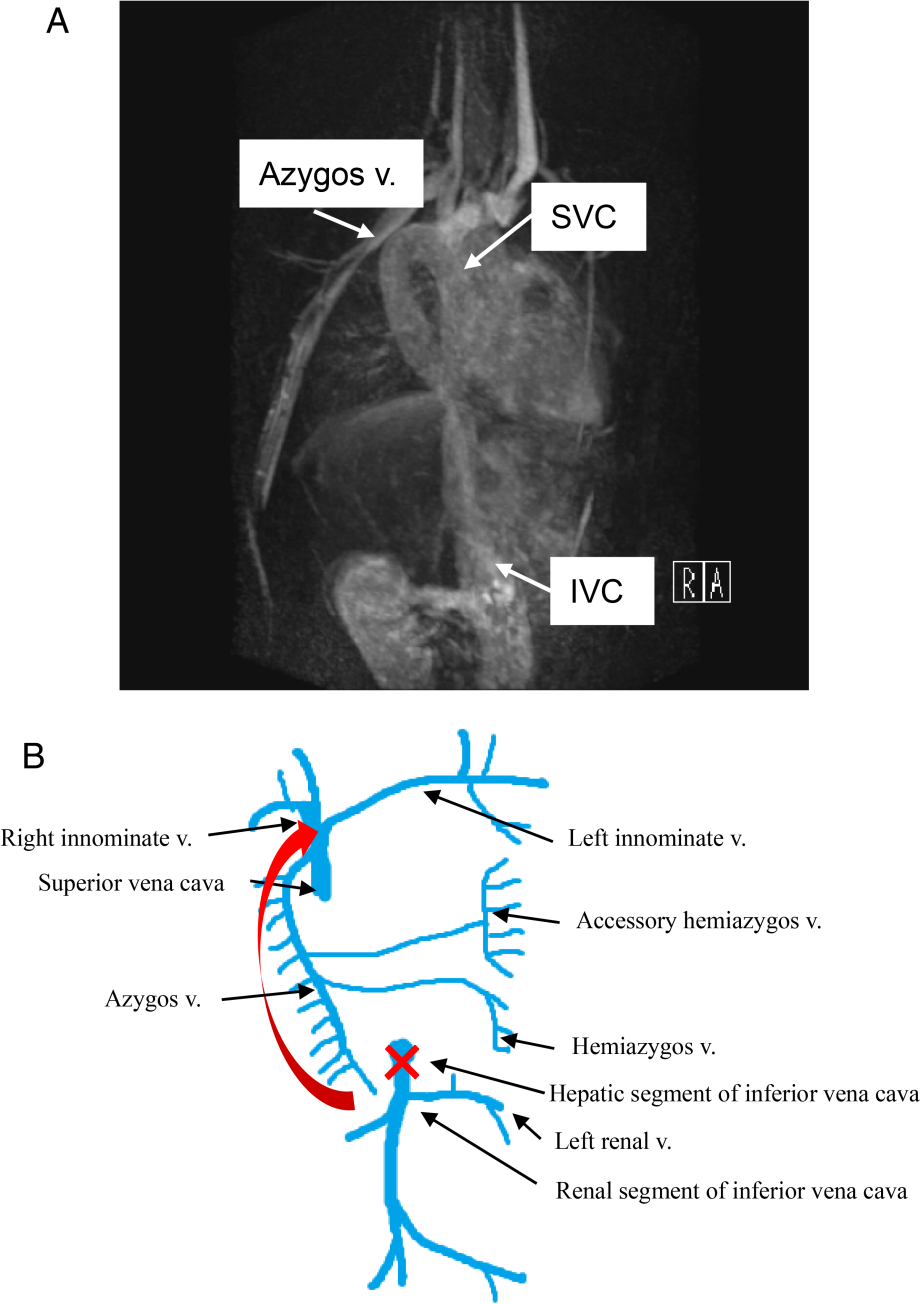
(A) Magnetic resonance angiography showed an inferior vena cava (IVC) connected to the superior vena cava (SVC) through the azygos vein. (B) Schema of venous flow in the azygos connection of the inferior vena cava.

A healthy 16‐year‐old male patient of mixed Japanese and Filipino descent presented with an abnormal shadow on a chest X‐ray taken during an annual check‐up. His family history was not remarkable. The patient had no past history of note. He was a non‐smoker. The chest X‐ray showed cardiomegaly and a falciform shadow in the right upper lobe angled towards the mediastinum, accompanied by an azygos lobe with a prominent fissure (Fig. 1). Chest computed tomography (CT) showed an enlarged azygos vein connecting to the inferior vena cava. There were no associated malformations. Magnetic resonance angiography showed that the inferior vena cava was connected to the superior vena cava via the azygos vein (Fig. 2A). Blood examination, including haemogram and tests of renal and hepatic function, was within normal limits. There were no abnormal findings on electrocardiography. Although managed with ongoing follow‐up alone, the patient is currently alive and well, and has been asymptomatic for the past two years.

## Discussion

The inferior vena cava is composed of a hepatic segment, a suprarenal segment, a renal segment, and an infrarenal segment. If the renal segment does not connect with the hepatic segment during the embryonic period (Fig. 2B, red cross), blood flow from the lower body drains into the superior vena cava through the right supracardinal vein (later, the azygos vein) later in life (Fig. 2B, arrow). This azygos connection of the inferior vena cava is reported to occur in 0.6% of patients with congenital heart disease [1]. Conventionally, azygos connection of the inferior vena cava was often reported to be associated with congenital abnormalities such as polysplenia, asplenia, atrial septal defect, total anomalous pulmonary venous connection, and heterotaxy. However, cases of azygos connection of the inferior vena cava without congenital abnormalities have also been reported, as with our case. This condition does not affect the patient's growth, and therefore observation alone will suffice. However, thoracic or abdominal surgery would be fatal if the flow through this connection was to be interrupted. There are some reports of successful surgery being performed for lung cancer or oesophageal cancer, being careful to protect the enlarged azygos vein where this condition is present [3]. Unfortunately, there is also a report of a patient who died owing to ligation of the azygos connection during surgery [4]. When interventional radiology such as ablation is performed in someone with this condition, it is important to note that an inferior vena caval approach fails to provide direct access to the atrium [5]. Therefore, to avoid fatal or inappropriate interventions, patients should be well informed about the presence of this malformation.

The azygos lobe is formed when migration of the azygos vein over the apex of the right lung towards the mediastinum is delayed during the embryonic period; the right upper lobe then becomes divided. Considered a normal variant, the azygos vein that runs through the azygos fissure is usually thin and not remarkable. The presence of an enlarged azygos vein in the azygos fissure indicates high flow through this vein. When an azygos lobe is identified on chest X‐ray, a prominent solid structure passing through it may represent an engorged azygos vein with an anomalous course.

### Disclosure Statement

Appropriate written informed consent was obtained for publication of this case report and accompanying images.
